# Retraction: Application of sewage sludge combined with thiourea improves the growth and yield attributes of wheat (*Triticum aestivum* L.) genotypes under arsenic-contaminated soil

**DOI:** 10.1371/journal.pone.0274852

**Published:** 2022-09-30

**Authors:** The PLOS ONE Editors

The *PLOS ONE* Editors retract this article [1] because it was identified as one of a series of submissions for which we have concerns about authorship, competing interests, and peer review. We regret that the issues were not addressed prior to the article’s publication.

NM, SK, SY, KtK, SAAlrumman, and EMA did not agree with the retraction. SFA responded but expressed neither agreement nor disagreement with the editorial decision. HS, SAMA, GM, SAAli, and SD either did not respond directly or could not be reached.
