# Correction: Retraction: Application of sewage sludge combined with thiourea improves the growth and yield attributes of wheat (*Triticum aestivum* L.) genotypes under arsenic-contaminated soil

**DOI:** 10.1371/journal.pone.0279581

**Published:** 2022-12-19

**Authors:** The PLOS ONE Editors

There is an error in paragraph 2, sentence 1, of this Notice of Retraction [1]. The correct sentence is: NM, SK, SY, KtK, SAAlrumman, and EME did not agree with the retraction.

The publisher apologizes for the error.
